# Assessing Current Therapeutic Approaches to Decode Potential Resistance Mechanisms in Glioblastomas

**DOI:** 10.3389/fonc.2013.00059

**Published:** 2013-03-19

**Authors:** Chun-I Sze, Wan-Pei Su, Ming-Fu Chiang, Chen-Yu Lu, Yu-An Chen, Nan-Shan Chang

**Affiliations:** ^1^Department of Anatomy and Cell Biology, College of Medicine, National Cheng Kung UniversityTainan, Taiwan; ^2^Institute of Molecular Medicine, National Cheng Kung UniversityTainan, Taiwan; ^3^Department of Neurosurgery, Mackay Memorial HospitalTaipei, Taiwan; ^4^Graduate Institute of Injury Prevention and Control, Taipei Medical UniversityTaipei, Taiwan; ^5^Advanced Optoelectronic Technology Center, National Cheng Kung UniversityTainan, Taiwan; ^6^Department of Neurochemistry, New York State Institute for Basic Research in Developmental DisabilitiesNew York, NY, USA; ^7^Department of Neuroscience and Physiology, State University of New York Upstate Medical UniversitySyracuse, NY, USA

**Keywords:** glioblastoma multiforme, temozolomide, resistance mechanisms, cancer stem cell, TIAF1 expression, extracellular matrix

## Abstract

Unique astrocytic cell infiltrating growth and glial tumor growth in the confined skull make human glioblastoma (GBM) one of the most difficult cancers to treat in modern medicine. Prognosis for patients is very poor, as they die more or less within 12 months. Patients either die of the cancer itself, or secondary complications such as cerebral edema, herniations, or hemorrhages. GBMs rarely metastasize to other organs. However, GBM recurrence associated with resistance to therapeutic drugs is common. Patients die shortly after relapse. GBM is indeed an outstanding cancer model to search for potential mechanisms for drug resistance. Here, we reviewed the current cancer biology of gliomas and their pathophysiological events that contribute to the development of therapeutic resistance. We have addressed the potential roles of cancer stem cells, epigenetic modifications, and epithelial mesenchymal transition (EMT) in the development of resistance to inhibitor drugs in GBMs. The potential role of TIAF1 (TGF-β-induced antiapoptotic factor) overexpression and generation of intratumor amyloid fibrils for conferring drug resistance in GBMs is discussed.

## Introduction

Glioblastoma (GBM) is one of the cancers most resistant to treatments and is associated with extremely poor prognosis. The current standard of care includes surgery with or without combined radiation/chemotherapy. Alternative treatment protocols and targeted therapies have been applied to GBM patients, but have unfortunately yielded to limited efficacies (Omuro et al., 2007; Lukas et al., 2009). Most cancer therapies have focused on attacking the key biological constituents that relate to the tumor growth and survival. It includes complex mechanisms of signal transduction and gene expression that contribute to the formation of microenvironment for supporting tumor growth (Omuro et al., 2007). Targeted therapies could be complicated by interconnecting signal networks with multiple interferences and convergences (Lukas et al., 2009). New methods and techniques for the targeted treatment of gliomas such as interruption of signaling pathways, nanoparticles targeting, and boron neutron capture therapy have been underway (Takagaki et al., 2001; Desai et al., 2006; Barth et al., 2012; Nduom et al., 2012). However, the clinical outcomes have made little progress in malignant gliomas to date (Omuro et al., 2007). It is thus necessary to reevaluate current strategies to find alternative approaches to eradicate malignant gliomas, or revisit the fundamental biology to explore the potential cancer resistance mechanisms in GBMs.

## GBM Classification and Common Markers

Histologically, GBMs can be derived from low-grade gliomas in younger patients, according to the classification of World Health Organization (WHO). Or, they can directly develop *de novo* in elderly patients (Ohgaki and Kleihues, 2007). Among the complex genetic pathways in the development of gliomas, oligodendrocyte transcription factor 2 (olig2) and vascular endothelial growth factor (VEGF) are expressed in all high-grade gliomas (Ohgaki and Kleihues, 2007). Therefore, classifying GBMs based on the activation of signal pathways or mutations of genes in the glioma-relevant pathways may help establish targeted therapies. For example, alterations in the activation of epidermal growth factor receptor (EGFR) and platelet-derived growth factor receptor (PDFGR), or loss of the RAS regulator NF1, are frequently observed in primary GBMs (Brennan et al., 2009). These findings implicate that these molecules as potential therapeutic targets. Although these classifications do not include all clinical GBMs, clinical trials targeting EGFR or PDFGR have been underway. EGFR, EGFR variant III (EGFRvIII), phosphatase, and tensin homolog deleted on chromosome 10 (PTEN), and O6-methylguanine–DNA methyltransferase (MGMT) have been regarded as common markers for GBMs (Camara-Quintana et al., 2012).

## Inhibitors of Receptor Tyrosine Kinases and Resistance in GBMs

Recent studies have shown that EGFR inhibitors fail to yield significant clinical outcomes in GBM patients. Simultaneous activation of multiple receptor tyrosine kinases (RTKs), which generates redundant activation of phosphoinositide-3′-kinase (PI3K) signaling, may explain for the drug failure (Fenton et al., 2012). Tumor suppressor PTEN, a phosphatidylinositol-3,4,5-trisphosphate 3 (PIP3) phosphatase, can be phosphorylated at a conserved tyrosine 240 (Y240). The phosphorylated PTEN (p-PTEN) is associated with shortened survival and resistance to therapy with EGFR inhibitors in GBM patients (Fenton et al., 2012). Both fibroblast growth factor receptors (FGFRs) and SRC family kinases (SFKs) phosphorylate PTEN, and p-PTEN fails to antagonize the PI3K signaling (Fenton et al., 2012), suggesting that loss of control of PI3K signaling is associated with resistance to EGFR inhibitors in GBM.

Amplification and/or mutation of a specific RTK gene in GBMs could confer resistance to RTK inhibitors. For example, genes encoding EGFR, platelet-derived growth factor receptor α (PDGFRα), hepatocyte growth factor receptor (MET), and/or others are frequently altered (Cancer Genome Atlas Research, 2008; Huse and Holland, 2010). It is not surprising to predict the failure in therapy using small-molecule inhibitors in targeting the mutated and/or amplified RTKs due in part to constitutive and concurrent activations of signal pathways in GBMs (Stommel et al., 2007; De Witt Hamer, 2010; Hasselbalch et al., 2010; Paulsson et al., 2011). Worse, growth factors could further enhance the drug resistance in subpopulations of GBM cells harboring amplifications of *EGFR* and *PDGFR*-*α* genes (Szerlip et al., 2012; Wilson et al., 2012). These observations highlight the role of RTK ligands and extensive redundancy of RTK-transduced signaling in innate and acquired resistance of GBMs to drugs targeting oncogenic kinases (Wilson et al., 2012).

## Cancer Stem Cells Confer Intrinsic Drug Resistance

Cancer stem cells (CSCs) are crucial in the initiation, progression, and angiogenesis for GBMs and essentially all cancers (Wen and Kesari, 2008; Dietrich et al., 2010). GBM CSCs express CD133 and nestin, which are also expressed by normal stem cells or progenitor cells. Whether drugs can specifically select against CSCs in the brain without affecting normal stem cells is not quite understood (Yilmaz et al., 2006; Calabrese et al., 2007). How CSCs develop into highly vascular GBMs is largely unknown. Expression of Olig2 and VEGF in all high-grade gliomas and glioma stem cells may render them highly vascular (Plate et al., 1992; Ohgaki and Kleihues, 2007; Takano, 2012). Anti-angiogenesis strategies to block CSC expansion have been utilized. However, the benefit of anti-angiogenesis therapy has been questionable in both preclinical and clinical trials.

The failure of anti-angiogenesis therapy may be due to evasive (adaptive) and/or intrinsic (pre-existing) resistance in GBM cells (Bergers and Hanahan, 2008). Simultaneous inhibition of cancer survival targets, along with potential escape pathways, may have a great potential in eliminating drug-resistant cancer cells. One such example is targeting both FGFRs and VEGF. Combined VEGF and FGFR–Fc fusion protein (FGF-trap) treatment attenuated revascularization and slowed tumor growth, which indicates that FGF signaling is involved in regulating angiogenesis (Casanovas et al., 2005). It has been reported that AZD2171, a pan-VEGF receptor tyrosine kinase inhibitor, normalizes tumor vasculature and alleviates cerebral edema in GBM patients. This study implies analogous evasive resistance may be mediated by FGF-dependent revascularization (Batchelor et al., 2007). Given that FGFRs also participate in EGFR and PDGFR-mediated cancer resistance, designing therapeutic regimes that simultaneously target activation-reactivation, and amplification of FGFRs and RTKs may be beneficial in resolving therapeutic resistance in GBM patients.

When tumors outgrow their blood supply, CSCs upregulate pro-inflammatory proteins to help tumor survival under hypoxic conditions (Tafani et al., 2011). In essence, CSCs play multi-faceted roles in allowing a tumor to escape complete eradication. Specific molecular markers that are capable of distinguishing CSCs from normal stem cells or progenitor cells in the brain is not completely resolved (Yilmaz et al., 2006; Calabrese et al., 2007). Therefore, before CSCs can be clearly identified, therapeutic approaches designated to target them may actually cause more harm than good to GBM patients.

## Epigenetic Modifications and GBM Resistance to Temozolomide

**Figure 1:**
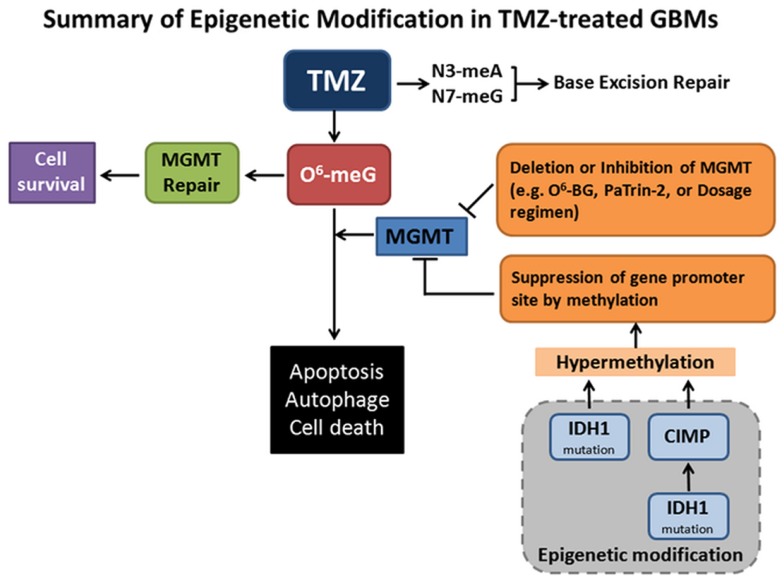
**Summary of epigenetic modifications in Temozolomide (TMZ)-treated GBMs**. TMZ is an oral alkylating agent used for the treatment of GBMs. TMZ causes cytotoxic DNA lesions such as O^6^-methylguanine (O^6^-meG), and N^3^-methyladenine and N^7^-methylguanine (N^3^-meA, N^7^-meG). The latter two lesions can be repaired by base excision repair (BER) pathway. O^6^-meG DNA methyltransferase (MGMT) removes the O^6^-alkylguanine and restores the guanine to normal, which promotes tumor cell survival. When MGMT transfers and accepts an alkyl-group from inhibitors O^6^-benzylguanine (O^6^-BG) and O^6^-(4-bromothenyl) guanine (PaTrin-2), MGMT is inactivated and subjected to ubiquitin-mediated degradation, thereby promoting cell death. MGMT functions may also be impaired by gene deletion, altered therapeutic regimen, and suppression of gene methylation promoter site. Notably, isocitrate dehydrogenase 1 (*IDH1*) mutation is the cause of CpG island methylator phenotype (CIMP) via stably reshaping the epigenome. *IDH1* mutation by itself is also sufficient to hypermethylate MGMT. MGMT hypermethylation causes DNA strand breaks, apoptosis, autophagy, and tumor cell death. This is the mechanistic rationale for the usage of the methylating therapeutic drugs.

Temozolomide (TMZ) with or without radiation is the current standard treatment for GBMs. Methylation of *MGMT* (O^6^-methylguanine–DNA methyltransferase) promoter prevents gene translation, and thereby prevents DNA repair in cancer cells. The methylation status of the *MGMT* promoter is currently considered one of the strongest predictors of outcome and benefit to TMZ treatment (Stupp et al., 2009). Mutant forms of metabolic enzyme isocitrate dehydrogenase (IDH1) are found in a great proportion of secondary gliomas, but are absent in the primary glioblastomas. The mutations are rarely found in primary high-grade glioblastoma multiforme. Clinical data have demonstrated that *IDH1* may be a reliable prognostic marker for GBM. Intriguingly, the presence of *IDH1* mutation(s) in patients with newly diagnosed GBMs showed prolonged, progression-free survival (Weller et al., 2009; Wick et al., 2009). GBMs with the CpG island methylator phenotype (CIMP) have been shown to possess extensive epigenetic aberrations to define a distinct subgroup of gliomas (Noushmehr et al., 2010). Recent data showed that *IDH* mutation and the CIMP phenotype are two very common features in cancer (Turcan et al., 2012). *IDH* mutation is the cause of CIMP and leads to the CIMP phenotype by stably reshaping the epigenome. This genome-wide remodeling involves modulating patterns of methylation, changing transcriptional programs, and alteration of cell differentiation (Turcan et al., 2012). These data highlight the interplay between genomic and epigenomic changes in human cancers and may prove to be helpful in the development of novel therapies for cancers. Shown in the Figure 1 is the summary of MGMT repair and epigenetic modification in TMZ treatment.

Epigenetic inactivation of tumor suppressor genes has been shown mainly in human malignant cancers. *DKK1* gene expression, for example, is decreased in GBM tumor samples, as compared with non-tumor brain tissues. Restoration of *DKK1* expression by a demethylating agent 5-azacytidine in T98 GBM cells enhances their susceptibility to camptothecin- and etoposide-induced apoptosis (Foltz et al., 2010), suggesting that treatment of GBM cells with inhibitors of promoter methylation for tumor suppressors facilitates drug efficacy. Also, regulatory enzymes for epigenetic alterations may be used for cancer therapy. When GBM cells were treated with histone deacetylase (HDAC) inhibitors, accumulation of methylated histone occurred. This histone methylation can be removed by lysine specific demethylase 1 (LSD1). Intriguingly, simultaneous inhibition of HDACs and LSD1 leads to synergistic apoptotic cell death in GBM cells (Singh et al., 2011).

## Epithelial Mesenchymal Transition and Drug Resistance in GBMs

Epithelial mesenchymal transition (EMT) is a critical biologic process that allows cancer cells to become metastatic. That is, there is an increased cell migratory capacity, invasiveness, and resistance to apoptosis (Kalluri and Neilson, 2003). EMT may occur in tumor cells that have previously undergone genetic and epigenetic changes, specifically in genes that favor clonal outgrowth, development of localized tumors, and enhancement of carcinoma invasion and metastasis (Jin et al., 2011). Growth factors are known to induce EMT include members of the EGF family, FGF, insulin-like growth factor, and MET (De Wever et al., 2008). Transforming growth factor beta (TGF-β) is released by glioma cells in large quantities *in vitro*. It has been implicated in the malignant progression of glial tumors and the immune dysfunction in patients with GBM (Xu et al., 2009). TGF-β promotes tumor-associated angiogenesis, tumor invasion, and suppression of T cell-mediated immune responses (Xu et al., 2009). Proto-oncogene *MET* (*c-met*) amplification in human gliomas causes increased activity of its downstream targets (e.g., Wnt/β-catenin signaling) (Moon et al., 2000; Kim et al., 2013), and prolongs the cancer survival (Halatsch et al., 2006). MET inhibitors appear to lead to aberrant *MET* amplification in gliomas (Moon et al., 2000; Chi et al., 2012). Dysregulation and prominent pathophysiological roles of EGFR have been identified in GBMs. EGFR is overexpressed in about 40% of GBM cases, and half of these co-express a mutated, activated subtype, EGFRvIII (Halatsch et al., 2006). Targeted therapy against EFGR and EGFRvIII has no survival benefit when compared to standard therapy. Stemness and invasiveness of migrating glioma cells regulated by *Frizzled 4* (*FZD4*), which promotes expression of the EMT transition regulator SNAI1, are considered as an important mechanism contributing to the failure of this approach (Jin et al., 2011; Pala et al., 2012). In its complexity, EMT encompasses pathways that promote the continuous acquisition of malignant biological features by glioma cells. A better understanding of the molecular mechanism of EMT may help identify and rationally design combined therapeutic regimes as well as selected patient subgroups that may benefit from EGFR inhibition in GBMs (Tektonidis et al., 2011; Pala et al., 2012).

## Potential Role of TIAF1 in GBM Progression

We have recently demonstrated that accumulation of extracellular proteins, which are generated by both cancer cells and neural cells, is critical for the self-protection, progression, and expansion of brain metastatic cancer cells (Lee et al., 2010; Chang et al., 2012). TGF-β-induced antiapoptotic factor (TIAF1) is an intracellular protein, whose self-aggregation intracellularly may induce generation of toxic amyloid beta (Aβ) and formation of amyloid fibrils (Lee et al., 2010; Chang et al., 2012). For example, when TIAF1 undergoes self-aggregation, the aggregating protein may stimulate caspase activation, subsequent phosphorylation of membrane amyloid precursor protein (APP), breakdown of APP, generation of toxic Aβ (e.g., Aβ42), and formation of amyloid fibrils (Lee et al., 2010; Chang et al., 2012). Aβ does not appear to cause cancer death. However, Aβ42 causes damage to neuronal death or induces neurodegeneration. TIAF1 is known to participate in the signaling of TGF-β/Smad proteins (Chang et al., 1998, 2012; Khera and Chang, 2003; Lee et al., 2010).

**Figure 2:**
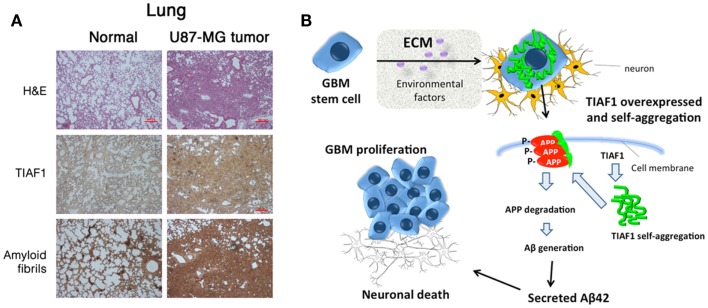
**A postulated model for GBM malignancy**. **(A)** U87-MG glioma cells were inoculated in both flanks of nude mice. Two months later, U87-MG cells were shown to metastasize to the lung. The solid tumor lesion has overexpressed TIAF1 and amyloid fibrils, compared to untreated normal lung. Specific antibodies against TIAF1 and amyloid fibril were used (Lee et al., 2010; Chang et al., 2012). **(B)** TIAF1 expression is frequently upregulated in the proliferating GBM cells, probably due to the stimulation of micro-environmental factors in the brain. The intracellular TIAF1 undergoes self-aggregation, may induce caspase activation, and leads to phosphorylation and degradation of membrane amyloid precursor protein (APP), generation of amyloid beta (Aβ), and formation of amyloid fibrils (Lee et al., 2010; Chang et al., 2012). Secreted Aβ42 is toxic to neurons. Also, Aβ42 undergoes self-polymerization and binds secreted TIAF1, and the complexes are probably detrimental to neurons. Aβ is indeed localized intracellularly. Cancer cells are resistant to the toxic effect of polymerized TIAF1 and Aβ. The presence of intracellular aggregated TIAF1 and amyloid fibrils in the tumor is likely to prevent the penetration and efficacy of therapeutic drugs.

TIAF1 is shown to be significantly upregulated in the malignant glioma cells in patients (Chang et al., 2012). A postulated model for GBM malignancy is that upregulation of TIAF1 occurs in the proliferating GBM stem cells, probably in response to extracellular stress (Figure 2). TIAF1 is then becoming self-aggregated intracellularly for leading to APP degradation and Aβ generation. Aβ is then released to the extracellular matrix, or polymerizes further to become amyloid fibrils, which are accumulated intracellularly. For example, when metastatic U87-MG glioma cells were inoculated in two subcutaneous sides in both flanks of nude mice, the cells were metastatic to the lung. Both aggregated TIAF1 and amyloid fibrils are overly expressed in the growing solid tumor (Chang et al., 2012) (Figure 2). The intracellular TIAF1 and amyloid fibrils may provide resistance to drug penetration into cancer cells, thereby promoting cancer cell survival.

## Conclusion

Human GBM is one of most challenging tumors to treat. Failure of therapeutic approaches to target specific signaling transduction or glioma-relevant mutation pathway genes in GBMs is mainly due to reactivation of certain common signaling pathways that are involved in cell growth and proliferation. Unpredictable clonal growth and specific gene amplification occur in CSCs. CSCs may also participate in treatment-induced evasive and/or intrinsic resistance and therefore therapeutic outcomes.

Ongoing “normal” epigenetic processes are associated with differentiation (Gan et al., 2007). Emerging data suggest that GBM patients with epigenetic MGMT methylation respond to TZM better and have prolonged survival than the patients without it. However, it is reported that patients without MGMT methylation may also be beneficial to TZM treatment (von Deimling et al., 2011), which suggests multiple mechanisms are involved in GBM pathogenesis. Direct genetic disruptions within the core epigenetic machinery may occur, such as the recently identified mutations within *IDH1/2* and variant histone genes *H3.3/H3F3A* (Carén et al., 2012). *IDH1* mutation alone is enough for the development of the glioma hypermethylate phenotype (Turcan et al., 2012). Therefore, *IDH1* may be used as a prognostic marker in low-grade and high-grade gliomas and aid in the differentiation and diagnosis of various tumors with histologic ambiguity. Insights into *MGMT*, *IDH1* mutation may help decode GBM resistance in both primary and secondary GBMs. EMT helps the continuous acquisition of malignant phenotype in GBMs, and may also contribute to the failure of targeted therapy.

In summary, development of GBM resistance involves multiple mechanisms with interplayed redundancies that determine the survival or death of cancer. Designing therapeutic regimes that target the aforementioned potential resistance mechanisms may likely be the primary focus of GBM treatment in the future.

## Authors Contribution

Wan-Pei Su, Chen-Yu Lu, and Yu-An Chen: worked on graphic arts, literature search, and proofread manuscript; Ming-Fu Chiang: proofread manuscript; Chun-I Sze and Nan-Shan Chang: wrote the manuscript, conceived ideas, and discussed thoroughly.

## Conflict of Interest Statement

The authors declare that the research was conducted in the absence of any commercial or financial relationships that could be construed as a potential conflict of interest.
